# Comparative genomics uncovered differences between clinical and environmental populations of *Vibrio parahaemolyticus* in New Zealand

**DOI:** 10.1099/mgen.0.001037

**Published:** 2023-06-02

**Authors:** Jack Vasey, Dan Jones, Cecilia H. Deng, Duncan Hedderley, Jaime Martinez-Urtaza, Andy Powell, Jing Wang, Jackie Wright, Anne-Marie P. Merien, Graham C. Fletcher, Sinisa Vidovic

**Affiliations:** ^1^​ The New Zealand Institute for Plant and Food Research Limited, Auckland, New Zealand; ^2^​ The New Zealand Institute for Plant and Food Research Limited, Palmerston North, New Zealand; ^3^​ School of Biosciences, Universitat Autonoma de Barcelona, 08193 Bellaterra, Spain; ^4^​ National Centre for Biosecurity and Infectious Disease, Centre for Environmental Fisheries and Agriculture Science, Weymouth, Dorset, UK; ^5^​ Institute of Environmental Science and Research Limited, Wellington, New Zealand; ^6^​ New Zealand Ministry for Primary Industries, Wellington, New Zealand

**Keywords:** *Vibrio parahaemolyticus*, pandemic strains, whole-genome sequencing, population genomics, non-synonymous mutations, virulence

## Abstract

*
Vibrio parahaemolyticus* has been identified as an emerging human pathogen worldwide with cases undergoing a global expansion over recent decades in phase with climate change. New Zealand had remained free of outbreaks until 2019, but different outbreaks have been reported consecutively since then. To provide new insights into the recent emergence of cases associated with outbreak clones over recent years, a comparative genomic study was carried out using a selection of clinical (mostly outbreak) and environmental isolates of *V. parahaemolyticus* obtained in New Zealand between 1973 and 2021. Among 151 isolates of clinical (*n*=60) and environmental (*n*=91) origin, 47 sequence types (STs) were identified, including 31 novel STs. The population of environmental isolates generated 30 novel STs, whereas only 1 novel ST (ST2658) was identified among the population of clinical isolates. The novel clinical ST was a single-locus variant of the pandemic ST36 strain, indicating further evolution of this pandemic strain. The environmental isolates exhibited a significant genetic heterogeneity compared to the clinical isolates. The whole-genome phylogeny separated the population of clinical isolates from their environmental counterparts, clearly indicating their distant genetic relatedness. In addition to differences in ancestral profiles and genetic relatedness, these two groups of isolates exhibited a profound difference in their virulence profiles. While the entire population of clinical isolates harboured the thermostable direct haemolysin (*tdh*) and/or the thermostable-related haemolysin (*trh*), only a few isolates of environmental origin possessed the same virulence genes. In contrast to *tdh* and *trh*, adhesin-encoding genes, *vpadF* and MSHA, showed a significantly (*P*<0.001) greater association with the environmental isolates compared to the clinical isolates. The effectors, VopQ, VPA0450 and VopS, which belong to T3SS1, were ubiquitous, being present in each isolate regardless of its origin. The effectors VopC and VopA, which belong to T3SS2, were rarely detected in any of the examined isolates. Our data indicate that the clinical and environmental isolates of *V. parahaemolyticus* from New Zealand differ in their population structures, ancestral profiles, genetic relatedness and virulence profiles. In addition, we identified numerous unique non-synonymous single-nucleotide polymorphisms (nsSNPs) in adhesins and effectors, exclusively associated with the clinical isolates tested, which may suggest a possible role of these mutations in the overall virulence of the clinical isolates.

## Data Summary

All of the assembled genomes were submitted to PubMLST genome collections (submission ID BIGSdb_20220902044256_007200_31649). Novel alleles and their DNA sequences were submitted to the PubMLST database. Further, the assembled genomes were submitted to the National Center for Biotechnology Information (NCBI) under the BioProject PRJNA952320.

**Impact Statement**

Human infections associated with *Vibrio parahaemolyticus* expanded globally during the last two decades, resulting in this organism becoming the leading cause of seafood infections worldwide. We characterized a population of environmental and clinical, mainly outbreak-associated, isolates of *V. parahaemolyticus* from New Zealand. The clinical isolates showed great homogeneity, distant genetic relatedness and different virulence profiles compared to the environmental isolates. The pandemic ST36 and outbreak ST50 isolates possessed a unique TDH allele and also various unique adhesins and effector alleles. Collectively, our results indicate that the majority of clinical isolates were not selected from the residential population of environmental isolates, but were introduced in New Zealand from somewhere else. More importantly, our study showed that the pandemic and outbreak isolates possess unique haemolysin, adhesin and effector alleles, suggesting a possible role of these mutations in the virulence or biological fitness of the pandemic ST36 and outbreak ST50 isolates.

## Introduction

*Vibrio parahaemolyticus *, a Gram-negative halophilic bacterium, is the leading cause of seafood-associated infections worldwide [1]. Interestingly, in many parts of the world, including Europe, North America, South America and India, this pathogen was associated with relatively rare human infections several decades ago. However, in the last two decades, human infections associated with *V. parahaemolyticus* expanded globally, affecting regions that previously had little or no incidence [2]. For instance, the Centers for Disease Control and Prevention estimated that during 2006–2017 the average annual incidence of vibriosis increased by 54 % in the USA, with *V. parahaemolyticus* being the most significant cause of this large increase [3]. The global dissemination of vibriosis caused by *V. parahaemolyticus* was associated with the emergence of strains with exceptional pandemic potential [2, 4–6]. The emergence of pandemic strains aligned with global warming, which most likely, together with better testing systems, contributed to the increased incidence rate [7]. The first recorded outbreak of *V. parahaemolyticus* in New Zealand took place in 2019 with the consumption of mussels contaminated with a pandemic ST-36 strain. The next two outbreaks of *V. parahaemolyticus* in New Zealand occurred consecutively in 2020 and 2021, causing further public health concerns in this country.

Generally, it is considered that strains of *V. parahaemolyticus* capable of producing thermostable direct haemolysin (TDH) and/or thermostable-related haemolysin (TRH) belong to a pathogenic population of this species. *V. parahaemolyticus tdh*+ and/or *trh*+isolates represent a minor subpopulation (0–6 %) in the overall population of *V. parahaemolyticus* [8–10], indicating that potential pathogenic strains are relatively rare in their natural niche.

Although the presence of two haemolysins, TDH and TRH, indicates an increased virulence potential, other virulence factors present in *V. parahaemolyticus* also contribute to the pathogenicity of this organism. Numerous reports show that isolates of *V. parahaemolyticus* without TDH and TRH can cause an invasion of the human host and develop a full infection [11–13], implying that the pathogenicity of this organism is more complex.

Besides haemolysins, *V. parahaemolyticus* possesses a wide range of adhesins that are not only involved in attachment, but also play a role in *V. parahaemolyticus *-mediated cytotoxicity. For instance, VpadF, a novel multiligand-binding adhesin, comprising an immunoglobulin-like group domain and a C-terminal tail, is capable of binding type I collagen, promoting a strong connection between *V. parahaemolyticus* and the host cell [14]. Deletion of the *vpadF* gene results in the significantly impaired adherence of *V. parahaemolyticus* to HeLa and HT-29 cell lines, reduced cytotoxicity and, finally, an attenuated virulence phenotype in the murine model [15]. This finding suggests an important role of VpadF adhesin in the overall pathogenicity of *V. parahaemolyticus *. Similarly, the mannose-sensitive haemagglutinin (MSHA), type IVb pili [16], and multivalent adhesion molecule 7 (MAM 7), a constitutively expressed adhesin [17], play essential roles in the attachment of *V. parahaemolyticus* and in penetration of the host barriers, aiding pathogen spread in host tissue [18, 19].

The pathogenicity of *V. parahaemolyticus* strains is closely associated with the existence of molecular syringes, sheath-tube nano-organelles that on contact puncture the host cell, allowing the injection of effectors (virulence-associated proteins) [20]. During host cell infection, *V. parahaemolyticus* activates type III secretion systems (T3SSs), specifically T3SS1. This system injects VopQ, VopS and VPA0450 into the host, the three main effectors, which are respectively involved in the prevention of phagocytosis [21], disruption of the host cell’s actin cytoskeleton [22], and disorganization of the host cell membrane [23]. T3SS1 is encoded in almost every isolate of *V. parahaemolyticus *, while the T3SS2 is sporadically present among the population of *V. parahaemolyticus* strains [24]. It is hypothesized that the T3SS1 plays a crucial role in the environmental survival of *V. parahaemolyticus* by lysis of host tissues, which provides growth support for this organism [25].

In this study, we examined the population structure, ancestral profiles, genetic relatedness and virulence profiles of a collection of environmental and clinical, mainly outbreak-associated, isolates of *V. parahaemolyticus* obtained from New Zealand, a geographically isolated region. We sought to identify unique mutations, non-synonymous single-nucleotide polymorphisms (nsSNPs), in three classes of virulence factors (toxins, adhesins and effectors) associated with environmental and clinical isolates of *V. parahaemolyticus *. In other words, we aimed to determine the polymorphic differences between these two groups of isolates that may contribute to the virulence associated with the clinical isolates.

## Methods

### Collection of *V. parahaemolyticus* isolates

In total, 152 *V *. *parahaemolyticus* isolates were collected. Of the 152 isolates, 92 were of environmental origin and 60 were from the faeces of clinical cases of gastroenteritis. All environmental isolates were obtained from New Zealand shellfish. Clinical isolates were received from the Institute of Environmental Science and Research (ESR), New Zealand with permission from the New Zealand Ministry of Health. The great majority of clinical isolates (*n*=54) were associated with the recent outbreaks that took place in New Zealand from 2019 to 2021. Two additional 2019 clinical isolates were from New Zealand cases who had recently travelled to Fiji, while another four clinical isolates were identified in New Zealand in 1973 [New Zealand Culture Collection (NZCC) NZRM3389], 1975 (NZCC NZRM3391), 2004 (NZCC NZRM4289) and 2013 (isolate H13ESR01658 – shared historically by ESR), respectively. These four isolates were not associated with an outbreak event. New Zealand has not had much history of gastroenteritis caused by *V. parahaemolyticus *, but all cases attributable to the organisms have been notifiable since 2017 [26]. Diagnostic laboratories (which use a variety of *Vibrio* testing methods) refer clinical isolates to the national reference laboratory at ESR for typing. Environmental isolates were mostly drawn from Plant and Food Research, a collection of New Zealand *V. parahaemolyticus* strains isolated between 2008 and 2013 during shellfish survey programmes by the Cawthron Institute [27] and Plant and Food Research [28]. A further seven environmental isolates were collected as part of the three outbreak investigations. Identification of clinical and environmental isolates of *V. parahaemolyticus* had been carried out using standard cultural and molecular methods [29]. Before use, each isolate was tested for its purity and stored at −80 °C in Luria–Bertani (LB) broth (Difco) with 10 % glycerol.

### DNA extraction and whole-genome sequencing

Genomic DNA of clinical isolates was extracted using the Chemagic 360 extraction platform (PerkinElmer, Waltham, MA, USA). The whole-genome libraries for clinical isolates were prepared using the Nextera XT DNA sample preparation kit (Illumina, San Diego, CA, USA). Paired-end sequencing of 2×150 bp was performed on the NextSeq 550 platform (Illumina, San Diego, CA, USA) at the ESR, Wellington, New Zealand. Genomic DNA of environmental isolates was extracted from overnight grown cultures using the DNeasy Blood and Tissue kit. The genomes of all the strains were sequenced by MiSeq with a coverage of 40–120× at the Centre for Environmental Fisheries and Agriculture Science, Weymouth, UK. Libraries were prepared with the Nextera XT DNA sample preparation kit (Illumina) and whole-genome sequence contigs for each strain were *de novo* assembled using the A5 pipeline [30]. Sequencing quality assessment and initial analysis, which included species identification, *de novo* assembly and multi-locus sequencing typing (MLST) assignment, were performed by the pipeline developed by the bioinformatics team of Plant and Food Research. The minimum coverage cut-off was 50 and the average quality score was at least 32.

### Genome assembly and gene identification

The full pipeline was implemented as an R Markdown document and is described in the accompanying bitbucket repository. In summary, read quality was assessed using FastQC 0.11.7/MultiQC 1.11. The genome of each isolate was assembled using Unicycler v0.4.8 with 2×150 bp paired-end Illumina sequencing reads. Genes in each genome were predicted using prokka 1.13. Independently of gene prediction, the nucleotide sequences corresponding to genes of interest (MLST loci and virulence genes) were identified within each genome by sequence homology using NCBI blast 2.11.0. The sequences were examined to ensure that the identified virulence genes were full length. Additionally, raw reads from each isolate were mapped to the genes of interest using Bowtie 2 2.3.4.3, to ensure that virulence genes that were absent from a genome were also absent from the raw reads used to assemble the genome.

### Population structure and ancestral source analyses

Sequence types (STs) of 151 environmental and clinical isolates were determined using the MLST method described by González-Escalona *et al*. [31]. One genome was dropped from further analysis due to poor quality. The nucleotide sequences corresponding to the MLST loci were concatenated into a single DNA sequence for each isolate. The allelic profiles and STs were determined by querying these concatenated sequences against the PubMLST database [31]. Novel alleles and their DNA sequences were submitted to the PubMLST database for the determination of new alleles’ numeric identifiers and STs. Ancestral profiles and clonal complexes of each identified genotype in this study were determined using a comparative eBURST [32] (Phyloviz 2.0) [33] approach, comparing already deposited worldwide data with the data from this study. For the determination of clonal complexes, the most stringent default group definition was used, where all STs have to be single-locus variants of at least one ST in the clonal complex.

### SNP calling and genome phylogeny

SNPs were called for the 151 isolates using bactsnp-1.1.0 pipeline [34] against the reference genome (RIMD 2210633). Data for each isolate were processed in parallel in a consistent way. The workflow combines two approaches in variant discovery, (i) assembly based and (ii) alignment based. To begin, raw sequencing reads from an isolate were trimmed with ‘platanus_trim’ (http://platanus.bio.titech.ac.jp/pltanus_trim). The cleaned reads were assembled into contigs using ‘platanus assemble’ (http://platanus.bio.titech.ac.jp/platanus-assembler/platanus-1-2-4). They were aligned to the reference genome using nucmer [35], and the assembly based pseudo-genome (ass_pseudo_genome.fa) was constructed using ‘delta2pseudo’ in bactsnp. Meanwhile, the trimmed reads were mapped to the reference genome using ‘bwa mem’ [36]. The alignments were sorted using ‘samtools sort’ [37], duplicates were marked using ‘MarkDuplicates’ module in Picard, and the cleaned alignments were indexed with ‘samtools index’. The mapping based pseudo genome (map_pseudo_genome.fa) was then built using ‘sam2pseudo’. Following that, the pseudo_genome for each isolate was created by merging the ass_pseudo_genome and map_pseudo_genome using ‘merge_fa’. SNPs were detected in all isolates and combined into a single tsv file using fa2snp. Finally, using snp2fa, the genome containing SNPs for each isolate was constructed and saved in the replaced_pseudo_genomes_wo_ref.fa (without the reference genome) and replaced_pseudo_genomes_w_ref.fa (with the reference genome) fasta files. The whole-genome SNPs phylogeny was inferred using the neighbour-joining method [38]. The percentage of replicate trees in which the associated taxa clustered together in the bootstrap test (1000 replicates) is shown next to the branches [39]. The evolutionary distances were computed using the maximum-composite-likelihood method [40]. All ambiguous positions were removed for each sequence pair (pairwise deletion option). The phylogenetic tree was created using mega11 [41].

### Presence–absence analysis of virulence encoding genes

In total, 13 virulence encoding genes, subdivided into 3 virulence classes, including toxins, adhesins and effectors (Table 1), were selected for this analysis. Besides two well-known haemolysin-encoding virulence genes, most of the known adhesin encoding genes of this organism were selected. Further, three well-characterized effectors associated with T3SS1 and T3SS2 were chosen for further analysis. The presence or absence of virulence-encoding genes was assessed in publicly available *V. parahaemolyticus* genomes from GenBank [42], and genomes from this study, as described in the above section. This included 151 genomes that were sequenced and assembled in this study. In addition, several reference genomes were used, including the following genome sequences RIMD 2210633, LVP2 and Vb0624. Full details of the genomes in this study are included in the Supplementary Material (Table S1, available in the online version of this article) and in the accompanying bitbucket repository. These sequences were examined to ensure that they were of the correct length for the corresponding virulence genes. Each isolate that showed a lack of the screened virulence sequence was examined again for the presence of the virulence gene using a general PCR assay (Table 1).

**Table 1.** Oligonucleotide primers used for the conformation of virulence genes

Gene	Protein function	Forward primer sequence (5’−3’)	Reverse primer sequence (5’−3’)	Amplicon size (bp)	Reference
*tdh*	Toxins	ATATCCATGTTGGCTGCATTC	TTATTGTTGATGTTTACATTCAAAA	531	[12]
*trh*		ATGAAACTAAAACTCTACTTTGC	TTAATTTTGTGACATACATTCAT	553	[12]
MAM 7	Adhesins	TAGCGGCGCGAACGTGCTCTAT	CCACGCCAGGCACGGAATCAAA	635	This study
*vpadF*		GATGCGATCACCAGTGCACCAT	ACCATCTTCATCACTCGCTACC	399	This study
*pilA*		CAACAGGGTTTCACTCTGATTG	ACCGTCGATAGAACTGTCTGCA	309	This study
MSHA		GAGTTAGTAGTGGTGATTGTGA	ATAGCTTTCGTTTGATGTAGTA	480	This study
*csgA*		ACAAGTGGCAGCATTTGCAGCA	CTGGTTAGCGGTGGCGTTGTTG	436	This study
orf8		GTTCGCATACAGTTGAGG	AAGTACACAGGAGTGAG	700	[63]
*vopQ*	Effectors	CGCTGCCATCAATTTGGCGTTA	CACCGTTCGCCGTAGATTCCGT	710	This study
*vopS*		CATCGTCTTCTGTCGAGCATGG	CGATGTGTTGCTTCAACATGCC	690	This study
Vpa0450		CAGCGACTTAAAGCTGAACTTG	ATCAACTTGTTGCCTTCGAACT	516	This study
*vopC*		CCGCGCTGAAAAGTGGTGATTT	TCTATTGCCGTGCATGTCATAC	388	This study
*vopA*		AGGATACAACCGCCAGATTTGT	CATCGCTTCCGGACGCGTTTCT	452	This study

### Analyses of premature stop codons and non-synonymous mutations

The nucleotide sequences of the virulence encoding genes were aligned using Clustal Omega [43] and examined for any premature stop codons by examining the protein translation of the gene. After this, the full-length gene sequences were imported into Geneious v10.2.51 [44]. Geneious was further used to translate each of these full-length genes into their corresponding protein sequences. The muscle2 alignment algorithm [45] within Geneious was used to perform multiple sequence alignments for the protein sequences of individual virulence factors. All resulting protein alleles were pooled.

## Results

### Population structure and ancestral profiles of the environmental and clinical isolates of *V. parahaemolyticus*

**Fig. 1.**
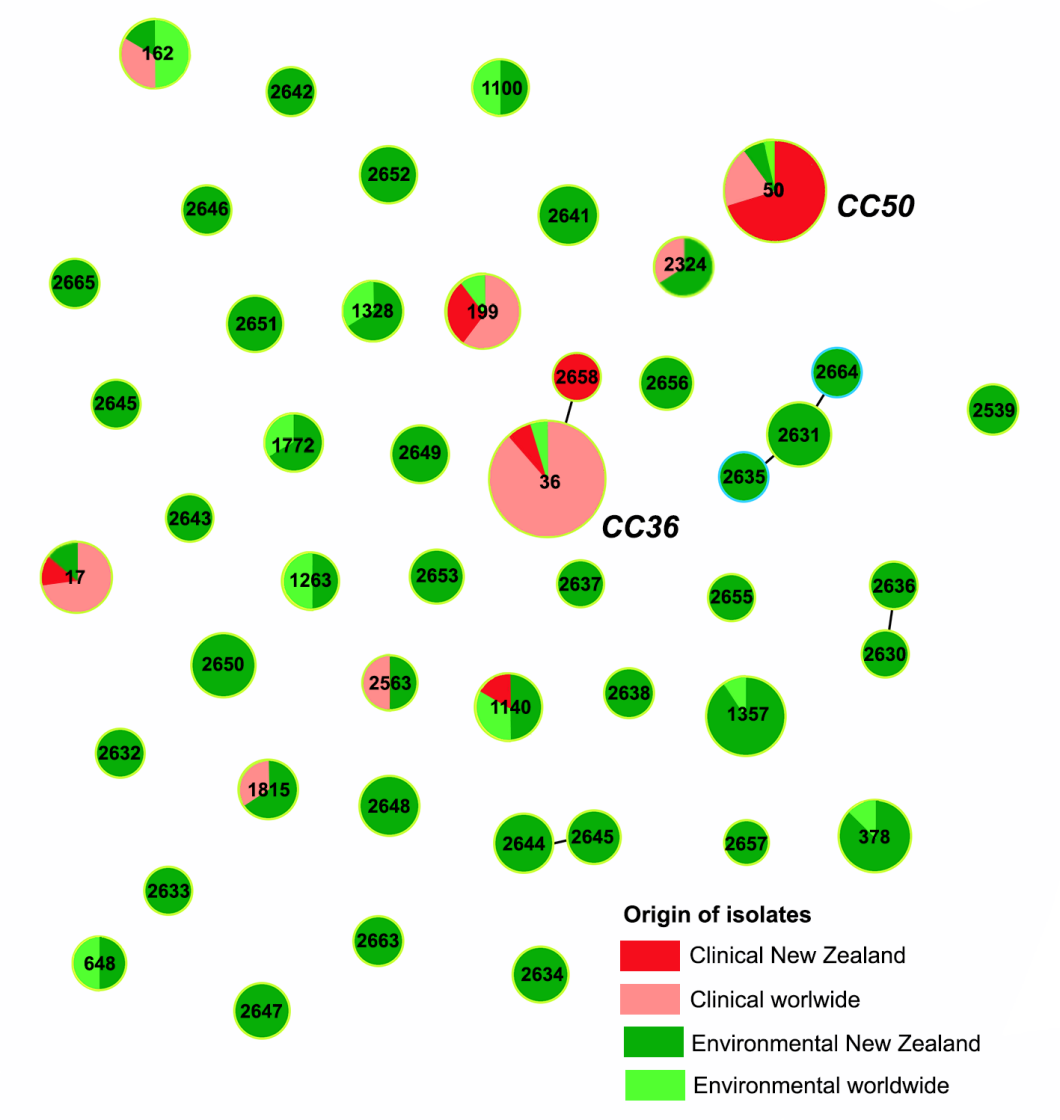
Population snapshot of environmental and clinical *V. parahaemolyticus* isolates isolated in New Zealand. All STs and two CCs identified in this study are numerically marked. Each dot represents a single ST, and the size of each dot is proportional to the number of isolates globally deposited. Sequence types not identified in this study have been removed from their clonal complexes for clarity.

In total, the 151 *V *. *parahaemolyticus* isolates were resolved into 47 STs (Fig. 1). One environmental isolate, ST2657 obtained in 2013 from Pacific oyster, was omitted from the dataset due to the poor quality of the SNPs. A great majority (87.2 %, *n*=41) of STs were associated exclusively with the environmental isolates, whereas only three STs, ST36, ST2658 and ST199, were exclusively associated with clinical isolates. Further, three STs (ST1140, ST17 and ST50) were found to be shared between both the environmental and clinical isolates of *V. parahaemolyticus* (Fig. 1), although the one ST50 detected in environmental samples was only detected when a lower detection limit (0.05 compared to 0.36 g^−1^) was applied to resolve an outbreak. Out of 47 STs, there were 31 previously unpublished, novel STs and only 1 of these was associated with the clinical isolates (ST2658). A total of 26 novel SNPs were identified among 7 MLST loci. The SNP density varied, ranging from the lowest SNP density of 458 (1 SNP per 458 nucleotides) in *dtdS* loci to the highest of 62 (1 SNP per 62 nucleotides) in *pyrC* loci. The population snapshot of all 151 environmental and clinical isolates, created by the comparative eBURST analysis, showed the existence of two clonal complexes (CCs), CC36 and CC50, which were predominantly associated with the isolates of clinical origin (Fig. 1). ST2658, the novel clinical ST, was a single-locus variant of ST36, harbouring a novel SNP in the *pyrC* loci.

Longitudinal data collected from 2008 to 2019 for the environmental isolates showed the existence of five spatially and temporally persistent STs, ST1357, ST378, ST2631, ST2650 and ST2648. ST1357 showed the most pronounced persistence, being isolated at six different geographical locations over 10 years, from 2009 to 2019. Among the novel STs, ST2631 showed the most noticeable persistence, being isolated at four distinct geographical locations from 2008 to 2013. Apart from these five spatially and temporally persistent STs, the great majority of the environmental isolates (24 STs) were only recorded once. All isolates with their IDs, accession numbers, STs, geographical location for environmental isolates, year of isolation and origins can be seen in Table S2.

### Genetic relatedness of the environmental and clinical isolates

**Fig. 2.**
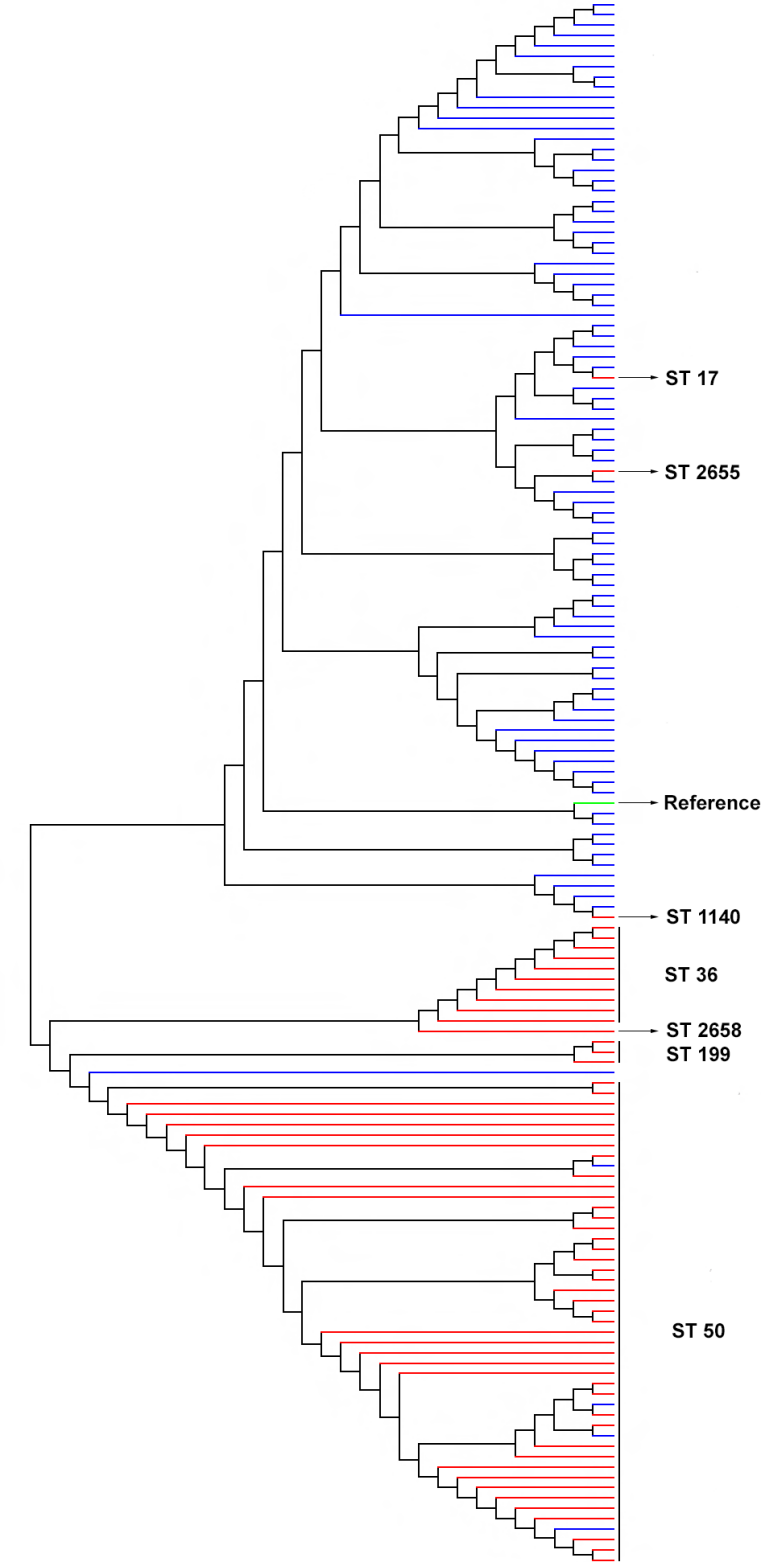
Phylogeny-based genome SNPs. The 150 clinical and environmental isolates of *V. parahaemolyticus* obtained from New Zealand were used to create a phylogenetic tree. The RIMD 2210633 genome of *V. parahaemolyticus* strain, isolated from Kansai region, Japan in 1996, was used as a reference genome. Red lines represent clinical isolates, blue lines represent environmental isolates and the green line indicates the reference isolate.

A total of 428 210 SNPs were used to create a neighbour-joining tree of the 151 isolates of clinical and environmental origins, including a reference RIMD 2210633 genome of *V. parahaemolyticus* (Fig. 2). The STs responsible for the majority of clinical cases in New Zealand (ST36 and ST50) fall into a clade that includes the clinical-associated STs 199 and 2658 as well as an environmental isolate of ST 2563. Within this clade each of the clinically associated STs forms a highly differentiated subclade (Fig. S1). All other isolates fall into a second clade dominated by environmental isolates but including clinical isolates of ST17 and ST1140. The overall average pairwise distance, an estimate of evolutionary divergence between sequences, for all clinical isolates was 0.0778, indicating that any two clinical isolates would diverge, on average, 7.78 % from each other based on their SNPs. The environmental isolates showed a strong genetic heterogeneity compared to their clinical counterparts, diverging from each other by 15.65 % based on their genome SNPs. In contrast, the smaller group of ST36 isolates (*n*=10) diverged from each other by 3.51 %, whereas the significantly larger group of the ST50 clinical isolates (*n*=47) showed profound genetic homogeneity, diverging from each other by only 0.015 %.

### Presence–absence variations of the virulence-encoding genes

**Fig. 3.**
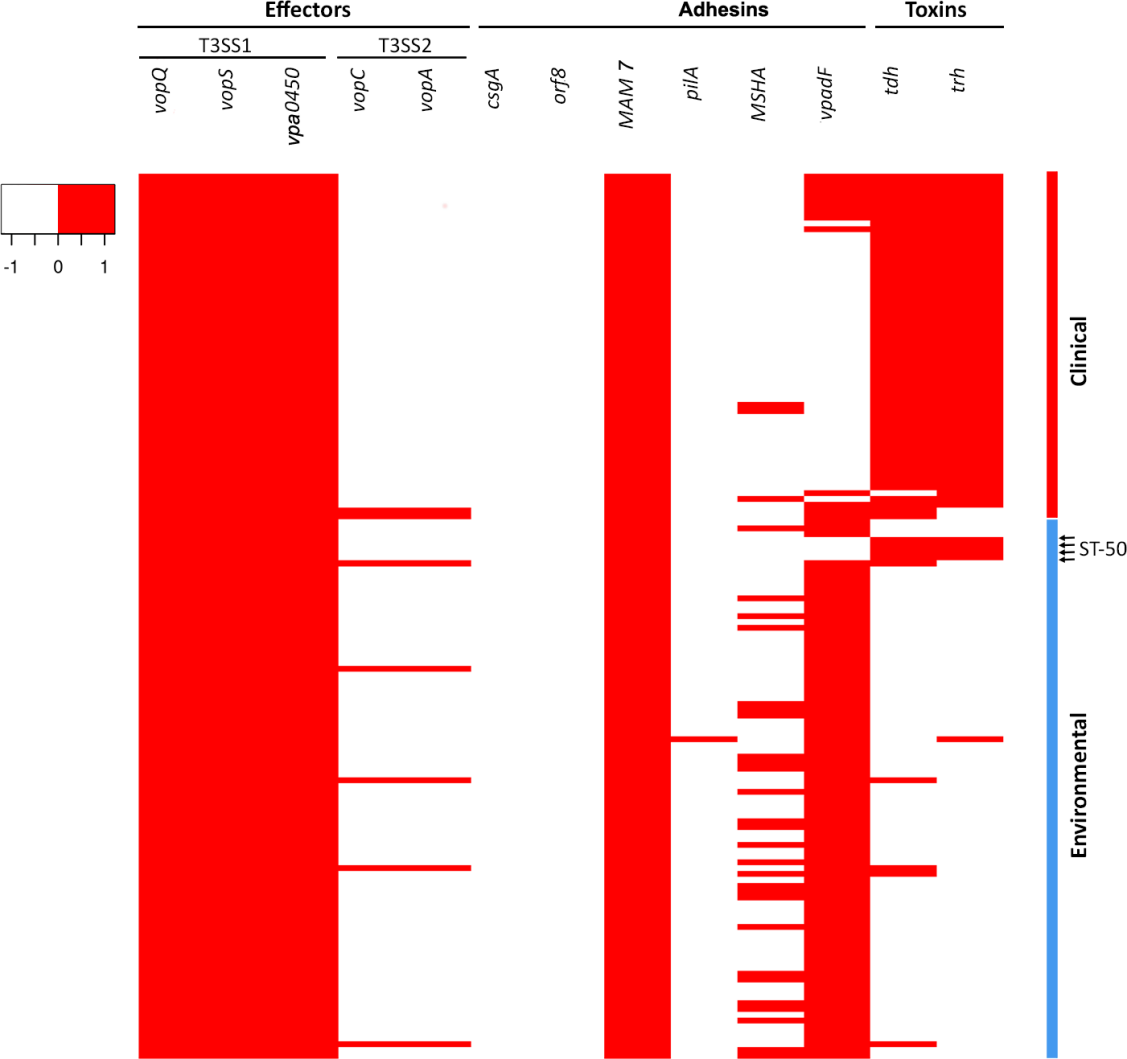
Virulence heat map. The figure shows the presence–absence variations of indicated effector-, adhesin- and toxin-encoding genes among the populations of the environmental and clinical isolates of *V. parahaemolyticus *. Presence is colour-coded: red indicates the presence of the virulence factors, while no colour indicates their absence.

To determine whether there is a difference in the virulence profiles of clinical and environmental isolates, the presence–absence variations of 13 virulence genes, including toxins, adhesins and effectors, were assessed out using the entire collection of *V. parahaemolyticus* isolates. Overall, there were three major patterns in the variations of virulence genes between the clinical and environmental groups: no difference, difference but not statistically significant and statistically significant difference (Table 2). Most virulence genes belonged to the first group, indicating a common virulence profile among the clinical and environmental isolates across virulence genes. All effectors associated with T3SS1, *vopQ*, *vopS* and *Vpa0450*, as well as adhesin *MAM 7*, were present in each isolate, whereas adhesins *csgA* and *orf8* were absent from each isolate (Fig. 3). The group of these virulence factors showed that the pathogen’s origin did not influence their occurrence in the tested population of *V. parahaemolyticus *.

Three virulence genes, including adhesin *pilA* and effectors associated with T3SS2, *vopC* and *vopA*, showed differences in their prevalence between the clinical and environmental isolates. However, these differences were not significant (Table 2), which also indicates that there is no relationship between the pathogen’s origin and the prevalence of these three virulence genes in the tested population.

There was a third group of virulence genes, including toxin-encoding genes *trh* and *tdh* as well as adhesin-encoding genes *vpadF* and MSHA, that were able to significantly differentiate the clinical isolates from their environmental counterparts (Table 2). A great majority (96.6 %) of the clinical isolates harboured the toxin-encoding gene, *tdh*, whereas only a minor portion (9.9 %) of the environmental isolates was positive for the same gene. Similarly, 95 % of the clinical isolates were positive for another toxin-encoding gene, *trh*, whereas 5.5 % of the environmental isolates were positive for the same gene (Table 2). All but one of the outbreak isolates harboured both toxin-encoding genes, *tdh* and *trh*, while all clinical isolates contained at least one of these two virulence factors (Fig. 3). Further, a group of four environmental isolates, all ST50, isolated from mussels during the outbreaks in 2020 and 2021, were also positive for both *tdh* and *trh* virulence factors. Six other environmental isolates were positive for either *trh* or *tdh* (Fig. 3).

In contrast to toxin-encoding genes, two adhesin-encoding genes, *vpadF* and MSHA, showed greater prevalence among the environmental isolates compared to their clinical counterparts (Table 2). The *vpadF* gene showed a strong discriminatory power between two groups of isolates, with 96.7 % of the environmental isolates being positive for this gene, whereas only 21.6 % of the clinical isolates harboured it (Table 2). The only environmental isolates lacking the *vpadF* gene were the four ST50 isolates obtained from mussels during the outbreaks of 2020 and 2021. They were epidemiologically linked to these outbreaks and were also positive for both toxin-encoding genes (Fig. 3). The MSHA gene was less prevalent in either the clinical or environmental isolates than the *vpadF* gene (Table 2).

**Table 2.** Presence–absence variations of virulence-associated genes among the populations of clinical and environmental isolates obtained from New Zealand

Virulence gene	No. (%) of isolates positive for the indicated virulence gene			
	Total (*n*=151)	Clinical isolates (*n*=60)	Environmental isolates (*n*=91)	Binomial generalized linear model, *P*
*tdh*	67 (44.3)	58 (96.6)	9 (9.9)	<0.001
*trh*	62 (41)	57 (95)	5 (5.5)	<0.001
MAM 7	151 (100)	60 (100)	91 (100)	1.000
*vpadF*	101 (66.8)	13 (21.6)	88 (96.7)	<0.001
*pilA*	1 (0.7)	0	1 (1.1)	0.313
MSHA	30 (19.9)	3 (5)	27 (29.7)	<0.001
*csgA*	0	0	0	1.000
*orf8*	0	0	0	1.000
*vopQ*	151 (100)	60 (100)	91 (100)	1.000
*vopS*	151 (100)	60 (100)	91 (100)	1.000
*vpa0450*	151 (100)	60 (100)	91 (100)	1.000
*vopC*	7 (4.6)	2 (3.3)	5 (5.5)	0.528
*vopA*	7 (4.6)	2 (3.3)	5 (5.5)	0.528

### Polymorphism in virulence-associated proteins

**Fig. 4.**
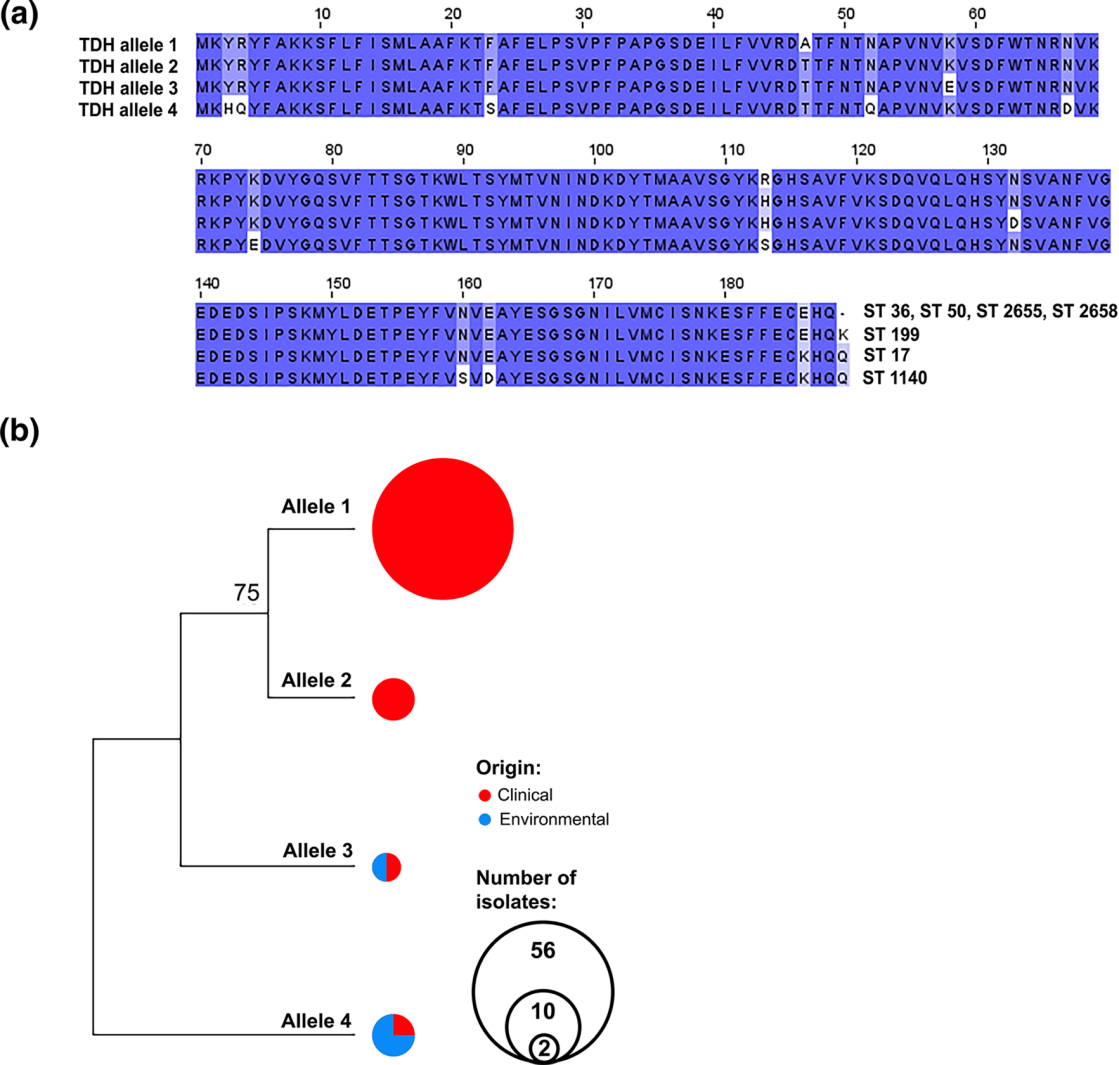
Polymorphism of TDH haemolysin. (**a**) Alignment of four TDH alleles found among the clinical and environmental isolates. Amino acids with no colour background represent unique amino acid substitutions. Association of STs with their TDH alleles is presented at the end of each allele sequence. (**b**) Phylogeny of the TDH alleles. Circle size represents the number of isolates associated with each allele.

No premature stop codon was detected in any of 879 protein sequences of 11 virulence factors derived from the population of environmental and clinical isolates of *V. parahaemolyticus *, indicating no existence of pseudogenes among the tested virulence factors. Two virulence factors, curli-specific gene A (*csgA*) and bacteriophage f237 (*orf8*), were absent from the entire population of *V. parahaemolyticus *. To determine polymorphism differences in the virulence factors between the clinical and environmental isolates, we identified all non-synonymous mutations. The total number of sequences, nsSNPs and protein alleles are shown in Table 3. In general, toxin-encoding genes (haemolysins) showed a low number of protein alleles compared to adhesins and effectors. Out of two haemolysins, only *tdh* harboured non-synonymous mutations, which further resulted in the generation of four TDH alleles. All pandemic (ST36), outbreak (ST50), ST2658 [single-locus variant (SLV) of ST36) and ST2655 isolates shared TDH allele 1. TDH allele 2 was associated with sporadic clinical ST199 isolates (Fig. 4a). Both TDH alleles 3 and 4 were associated with a small population of clinical and environmental isolates of ST17 and ST1140, respectively (Fig. 4a, b). TDH allele 1 harboured two unique amino acid substitutions, T47A and HS113R, whereas TDH allele 2 had no unique non-synonymous mutations. TDH allele 4 exhibited nine unique amino acid substitutions, while TDH allele 3 harboured two unique amino acid substitutions, K58E and N132D (Fig. 4a). TDH alleles 1 and 2, which were exclusively derived from clinical isolates, showed a close genetic relatedness compared to that of the TDH alleles 3 and 4, which were a combination of clinical and environmental isolates (Fig. 4b).

**Table 3.** Determination of protein alleles

Protein	Total no. of sequences	No. of polymorphic sites	No. of protein alleles
Thermostable direct haemolysin	64	14	4
Tdh-related haemolysin	59	0	1
Multivalent adhesion molecule 7	151	25	28
* V. parahaemolyticus* adhesive factor	96	94	40
Chitin-regulated pilus A	1	0	1
Mannose-sensitive haemagglutinin	30	6	7
* Vibrio* outer protein Q	151	74	35
Inositol polyphosphate-5-phosphatase	151	26	27
* Vibrio* outer protein S	151	34	30
* Vibrio* outer protein C	7	3	3
* Vibrio* outer protein A	7	3	3

**Fig. 5.**
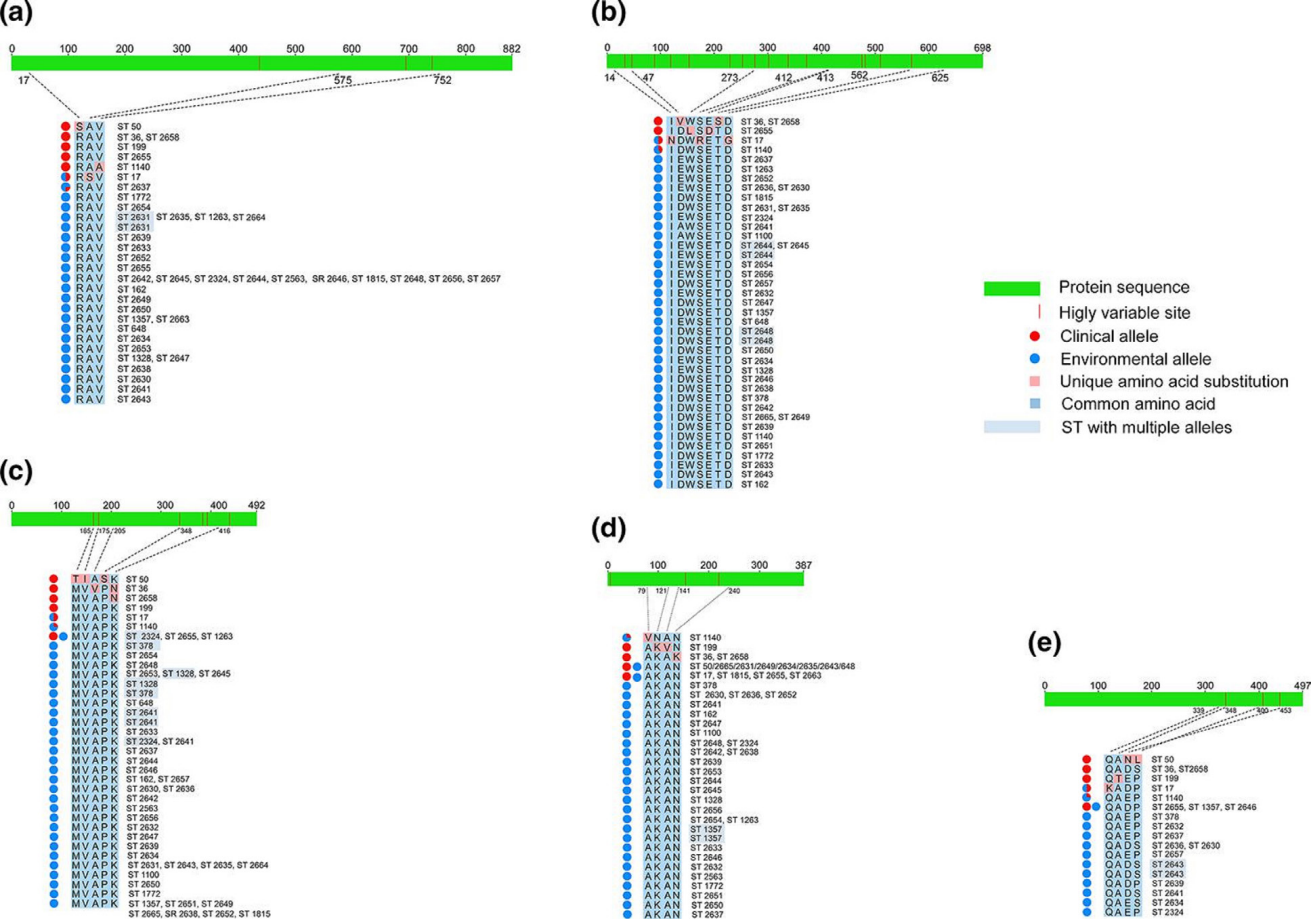
Unique nsSNPs in adhesin and effector alleles of clinical isolates. The length of each adhesin and effector is indicated above the protein sequence. Positions of unique nsSNPs in clinical alleles are shown by dotted lines. All identified clinical and environmental alleles and their associations with STs are shown. (**a**) Allelic profile of adhesin MAM 7. (**b**) Allelic profile of adhesin VpadF. (**c**) Allelic profile of effector VopQ. (**d**) Allelic profile of effector VopS. (**e**) Allelic profile of effector VPA0450.

In contrast to haemolysins, where the single allele was shared among most clinical isolates regardless of ST, clinical alleles of adhesins were specifically associated with a single ST. In other words, adhesin alleles derived from the clinical STs exclusively contained single ST or a ST and its SLV. The environmental STs showed a less stringent pattern of mutation distribution compared to that of the clinical isolates. Among the population of environmental isolates, different STs shared the same adhesin allele or a single ST possessed multiple adhesin alleles (Fig. 5a, b). Multiple protein alignments of MAM 7 and VpadF revealed the existence of 3 and 15 non-conserved sites within these 2 adhesins, respectively (Fig. 5a, b). Three unique amino acid substitutions, S17R, S575A and A752V, distinguished MAM 7 adhesin alleles associated with the isolates of outbreak ST-50, clinical ST17 and ST1140, respectively, from the rest of MAM 7 alleles (Fig. 5a). The MAM 7 allele of pandemic ST36 did not harbour any unique non-synonymous mutation but rather it contained a unique combination of already present amino acid substitutions. In contrast to MAM 7, the VpadF allele associated with pandemic ST36 and its SLV ST2658 harboured two unique mutations, V47D and S502T, separating isolates of this pandemic strain and its SLV from the rest of *V. parahaemolyticus* population (Fig. 5b). The other two VpadF alleles associated with the clinical isolates, ST2655 and ST17, also contained unique amino acid substitutions (Fig. 5b).

Each effector, VopQ, VopS and VPA0450, contained three non-conserved sites (Fig. 5c–e). The clinical isolates of pandemic ST36, its SLV 2658, and outbreak ST50 harboured several unique non-synonymous mutations in the effector VopQ, while other clinical STs acquired unique combinations of the common mutations, further resulting in distinct clinical VopQ alleles (Fig. 5c). Only clinical ST2655 shared the same VopQ allele with two environmental STs, ST1357 and ST2646 (Fig. 5c). The effector VopS was characterized by a low number of clinical alleles. Isolates of ST1140, ST199, ST36 and its SLV ST2658 contained distinct alleles of clinical origin, whereas clinical isolates of ST50, ST17 and ST2655 shared their VopS alleles with numerous environmental STs (Fig. 5d). Similar to VopQ, effector VPA0450 was characterized by a great separation between clinical and environmental STs. All clinical STs were associated with distinct VPA0450 alleles, whereas only clinical isolates of ST2655 shared the VPA0450 allele with other two environmental STs, ST1357 and ST2646 (Fig. 5e).

## Discussion

The present population-based study revealed a profound heterogeneity of the environmental isolates, where 91 isolates generated 41 different STs. In contrast to the environmental isolates, the mostly outbreak-associated population of clinical isolates (*n*=60) showed great homogeneity, resulting in only six STs. Besides the difference in the population structure between the clinical and environmental isolates, we observed a significant (*P*<0.001) difference in the ancestral profiles between these two groups of isolates. Out of 41 STs associated with the environmental isolates, 31 (75.6 %) were novel, whereas, of the 6 STs of the clinical origin, only 1 (16.6 %) was novel. The novel clinical ST (ST2658) was a SLV of the pandemic ST36 strain, indicating further evolution of this pandemic strain. The novel ST was isolated from a case of gastroenteritis in Southland, New Zealand in 2013, suggesting that a strain that shares a recent evolutionary history with the ST36 pandemic strain might have been present in New Zealand several years before the first reported outbreak in 2019. Taken together, our results suggest the existence of a diverse population of environmental *V. parahaemolyticus* isolates, unique to New Zealand, and the introduction of the strains with clinical background into the marine environment of New Zealand. The main line of evidence for this assumption lies in the fact that all clinical STs identified in New Zealand, except for ST2658, a minor SLV of ST36, have been already reported, mainly as the causative agents of vibriosis in human patients worldwide (http://pubmlst.org/vparahaemolyticus) [31, 46–48]. In other words, the clinical isolates were likely not selected from the population of residential environmental isolates of New Zealand but rather introduced to the marine environment of New Zealand from somewhere else.

In the past decade, numerous comparative genomics-based studies [49–52] have used populations of clinical and environmental *V. parahaemolyticus* isolates in attempts to uncover the critical virulence factors that distinguish the sub-population of clinical isolates from their environmental counterparts. In this study, examining a geographically defined collection of 151 clinical, mainly outbreak-related, isolates from 2019, 2020 and 2021, and environmental isolates derived from New Zealand, it was found that the haemolysin-encoding genes, *tdh* and *trh*, were strongly associated with the clinical isolates, but not with their environmental counterparts.

There are conflicting reports in the literature regarding the associations of the major virulence factors, TDH and TRH, with isolates of clinical and environmental origin. For instance, Jones *et al.* [53] observed that 27 % of all clinical strains collected across North America from July 2006 to November 2007 were negative for both TDH and TRH virulence factors. In contrast, Xie and colleagues [51], examining the clinical and seafood-associated isolates from Guangdong, PR China, found that the clinical isolates were 100 and 77% positive for *tdh* and *trh*, respectively, whereas the seafood isolates contained only 2.1 and 28.4 %, respectively, of the same virulence-associated genes.

The differences in the prevalence of *tdh* and *trh* among the clinical isolates can be explained by coinfection [54], loss of these virulence factors during infection [50], or simply by the existence of uncharacterized virulence factors in *tdh-* and *trh*-negative clinical isolates [50]. The high prevalence of the *tdh* and *trh* genes among the New Zealand clinical isolates can be explained by the fact that the great majority of these clinical isolates belong to two outbreak strains, ST36 and ST50, both *tdh-* and *trh*-positive.

Besides haemolysins, we observed that the adhesin-encoding genes, *vpadF* and MSHA, were not evenly distributed between environmental isolates and their clinical counterparts. Both of these adhesins showed a strong association with the environmental isolates compared to the clinical isolates. Again, this difference was strain-dependent, as all ST50 isolates lacked the *vpadF* gene, but other clinical STs, including ST36, ST17, ST1140 and ST2658, possessed this gene. Our study also showed the ubiquitous nature of the effector-encoding genes associated with T3SS1, regardless of the origin of isolates. In contrast to the effectors of T3SS1, we observed a significantly low prevalence of the effectors associated with T3SS2. This feature of New Zealand isolates differed from the environmental and clinical isolates of the Pacific Northwest (PNW) [55]. It was shown that T3SS2-associated genes were widespread in the PNW population, further indicating the existence of a distinctive population of *V. parahaemolyticus* in New Zealand.

It has been shown that point mutations in various proteins, including adhesins, toxins and efflux pumps, can lead to significant changes in the biological fitness of microbial organisms, further resulting in the emergence of highly virulent [56–59] or multidrug-resistant pathogens [60, 61]. To determine whether there are unique nsSNPs in haemolysins, adhesins and effectors associated with the population of clinical isolates, we carried out comparative genomics on the entire collection of *V. parahaemolyticus* isolates, testing selected haemolysins, adhesins and effectors.

Although there was a significant association of the haemolysin (*tdh*-)encoding gene with the clinical isolates, we observed further segregation of the population of *V. parahaemolyticus* harbouring this gene. The most notable difference was a complete association of all isolates of the dominant STs (pandemic ST36, outbreak ST50) with TDH allele 1, indicating the possible existence of a more virulent form of TDH allele.

The phylogeny of the TDH alleles clearly showed the close relatedness among the STs, exclusively associated with human infections compared to the minor clinical STs shared between the environmental and clinical isolates. This finding further indicates the possible role of nsSNPs in the evolution of pathogenic traits associated with TDH haemolysin. Unfortunately, our study, based on comparative genomics, cannot provide definitive confirmation of this hypothesis, but it would be worth determining the virulence potency of different TDH alleles in an animal model.

The population of clinical isolates harboured numerous unique nsSNPs or exhibited a unique combination of nsSNPs, which resulted in the generation of distinct clinical alleles and a clear separation of clinical isolates from their environmental counterparts. In the case of the VopS effector, we observed that certain clinical isolates shared their alleles with the environmental isolates. In all other cases, there was a clear segregation between the clinical and environmental isolates based on their adhesin and effector alleles. Unlike TDH haemolysin, where all clinically dominant STs shared the same allele, in adhesins (VpadF, MAM 7) and effectors (VopQ, VPA0450) the dominant STs had different alleles. In a part, this could be explained by the functional constraint of TDH. However, both, VopQ and VPA0450 effectors have specific roles, including repression of host cell energy metabolism [62] and disruption of host cell membrane integrity [23], respectively, which implies that these virulence factors are under similar functional constraints to TDH. Again, it would be important to elucidate the binding affinity and virulence potency of distinct clinical alleles of tested adhesins and effectors, which could lead to a better understanding of the pathogenicity of certain strains enabling them to cause outbreaks over a long period at various geographical locations.

In summary, using a temporally and geographically defined population of clinical and environmental isolates, in conjunction with MLST, comparative eBURST analysis and genome phylogeny, we showed that the clinical and environmental isolates of *V. parahaemolyticus* from New Zealand differ in their population structures and ancestral profiles and have distant genetic relatedness. Furthermore, these two groups of isolates showed profound differences in their virulence profiles, including haemolysin-encoding genes, *tdh* and *trh*, as well as adhesin-encoding genes, *vpadF* and MSHA. By performing comparative genomic analysis on the major virulence factors (haemolysin, adhesins and effectors), we identified numerous unique nsSNPs, exclusively associated with either the clinical or environmental isolates, suggesting a possible role of these nsSNPs in the virulence or environmental survival of the clinical and environmental isolates, respectively.

## Supplementary Data

Supplementary material 1
