# Supplementary material for: Comparative genomics uncovered differences between clinical and environmental populations of Vibrio parahaemolyticus in New Zealand
Source: Microb Genom. 2023 Jun 2;9(6):mgen001037. doi: 10.1099/mgen.0.001037 (PMC10327497; doi:10.1099/mgen.0.001037)
Supplement: Supplementary material 1 [file mgen-9-1037-s001.pdf]

Table S1. Reference Sequences Used in this Study.

Virulence Gene + Reference Organism*	GenBank Genome Assembly Accession Number	Locus Tag of Virulence Gene
tdh[1]	GCA_000196095	VP_RS21465
trh[2]	GCA_001011015	WU75_RS11390
MAM 7[1]	GCA_000196095	VP_RS07755
vpadF[1]	GCA_000196095	VP_RS08500
pilA[1]	GCA_000196095	VP_RS12235
MSHA[1]	GCA_000196095	VP_RS18765
csgA[3]	GCA_000978665	XU19_20665
orf8[1]	GCA_000196095	VP_RS07535
vopQ[1]	GCA_000196095	VP_RS08095
VPA0450[1]	GCA_000196095	VP_RS17390
vopS[1]	GCA_000196095	VP_RS08115
vopC[1]	GCA_000196095	VP_RS21495
vopA[1]	GCA_000196095	VP_RS21610
vopV[1]	GCA_000196095	VP_RS21665

*Reference organism from which the reference virulence gene sequence was extracted is denoted via superscript: [1] = *V. parahaemolyticus* RIMD2210633. [2] = *V. parahaemolyticus* ATCC17802. [3] = *V. parahaemolyticus* Vp103

Table S2. List of environmental and clinical isolates of *V. parahaemolyticus* originating from New Zealand and Fiji. (NZCC = New Zealand Culture Collection)

#	Isolate name	PubMLST accession number	ST	Geographic location	Country	Isolation year	Source
**1**	A11284_1 (PFR21B03)	6294	1140	Hauraki Gulf	NZ	2009	Pacific oyster
**2**	A11285-1 (PFR21C02)	4074	2631	Bay of Islands	NZ	2009	Pacific oyster
**3**	A11286-1 (PFR21C03)	4075	2632	Bay of Plenty	NZ	2009	Pacific oyster
**4**	A11287-1 (PFR21C04)	4088	2641	Bay of Plenty	NZ	2009	Pacific oyster
**5**	A11288_1 (PFR21D01)	4089	2641	Bay of Plenty	NZ	2009	Pacific oyster
**6**	A11289_1 (PFR21E06)	6299	2324	Bay of Plenty	NZ	2009	Pacific oyster
**7**	A11290_1 (PFR21E09)	6300	1357	Whangarei	NZ	2009	Pacific oyster
**8**	A11291-1 (PFR21F03)	4076	2631	Whangaroa Harbour	NZ	2009	Pacific oyster
**9**	A11292-1 (PFR21G03)	4090	2642	Kaipara Harbour	NZ	2009	Pacific oyster
**10**	A11293_1 (PFR21G10)	6303	1357	Bay of Islands	NZ	2009	Pacific oyster
**11**	A11294-1 (PFR21H05)	4091	2641	Bay of Plenty	NZ	2009	Pacific oyster
**12**	A11295-1 (PFR21H08)	6305	2643	Bay of Islands	NZ	2009	Pacific oyster
**13**	A11296-1 (PFR21H10)	4077	2631	Kaipara Harbour	NZ	2008	Pacific oyster
**14**	A11297-1 (PFR21I02)	4093	2644	Bay of Islands	NZ	2009	Pacific oyster
**15**	A11298_1 (PFR21I07)	4078	2633	Whangarei	NZ	2009	Pacific oyster
**16**	A11299-1 (PFR21I10)	4094	2645	Bay of Islands	NZ	2009	Pacific oyster
**17**	A11300 (PFR21J02)	4129	2663	Hauraki Gulf	NZ	2008	Pacific oyster
**18**	A11301-1 (PFR21J04)	4095	2645	Bay of Islands	NZ	2008	Pacific oyster
**19**	A11302-1 (PFR21J05)	4096	2646	Bay of Plenty	NZ	2008	Pacific oyster
**20**	A11303_1 (PFR21J08)	6313	2563	Whangaroa Harbour	NZ	2009	Pacific oyster
**21**	A11304-1 (PFR22C05)	4079	2634	Whangarei	NZ	2009	Pacific oyster
**22**	A11305-1 (PFR22E07)	4097	2647	Whangaroa Harbour	NZ	2008	Pacific oyster
**23**	A11306-1 (PFR22F01)	4080	2635	Whangaroa Harbour	NZ	2009	Pacific oyster
**24**	A11307_1 (PFR22G10)	6317	1263	Bay of Islands	NZ	2009	Pacific oyster
**25**	A11308_1 (PFR22H01)	6318	1328	Bay of Plenty	NZ	2009	Pacific oyster
**26**	A11309-1 (PFR24A01)	4098	2654	Coromandel	NZ	2010	Pacific oyster
**27**	A11310_1 (PFR24B03)	6320	1100	Kaipara Harbour	NZ	2010	Pacific oyster
**28**	A11311-1 (PFR24B07)	4099	2651	Coromandel	NZ	2010	Mussels
**29**	A11312-1 (PFR24B10)	4100	2652	Hauraki Gulf	NZ	2010	Pacific oyster
**30**	A11313_1 (PFR24C07)	4081	2634	Kaipara Harbour	NZ	2010	Pacific oyster
**31**	A11314-1	4101	2655	NZRM 4289	NZ	2004	Clinical
**32**	A11315-1 (PFR24E04)	4102	2649	Kaipara Harbour	NZ	2010	Pacific oyster
**33**	A11316_1 (PFR24E10)	6326	378	Hauraki Gulf	NZ	2010	Pacific oyster
**34**	A11317_1 (PFR24F07)	6327	1357	Coromandel	NZ	2010	Pacific oyster
**35**	A11318-1 (PFR24F10)	4103	2653	Kaipara Harbour	NZ	2010	Pacific oyster
**36**	A11319-1 (PFR24G05)	4104	2650	Coromandel	NZ	2010	Mussels

Table S2. Continued.

#	Isolate name	PubMLST accession number	ST	Geographic location	Country	Isolation year	Source
**37**	A11320_1 (PFR24G08)	6330	1815	Coromandel	NZ	2010	Pacific oyster
**38**	A11321_1 (PFR24H01)	6331	17	Bay of Islands	NZ	2010	Pacific oyster
**39**	A11322_1 (PFR24H02)	4105	2651	Hauraki Gulf	NZ	2010	Pacific oyster
**40**	A11323-1 (PFR24H05)	6333	162	Mahurangi Harbour	NZ	2010	Pacific oyster
**41**	A11324_1 (PFR24H08)	6334	1772	Hauraki Gulf	NZ	2010	Pacific oyster
**42**	A11325_1 (PFR24I08)	6335	1815	Hauraki Gulf	NZ	2010	Pacific oyster
**43**	A11326-1 (PFR24J01)	4106	2644	Kaipara Harbour	NZ	2010	Pacific oyster
**44**	A11327-1 (PFR24J05)	4107	2647	Hauraki Gulf	NZ	2010	Pacific oyster
**45**	A11328_1 (PFR24J06)	6338	1357	Hauraki Gulf	NZ	2010	Pacific oyster
**46**	A11329_1 (PFR24J08)	6339	1357	Mahurangi Harbour	NZ	2010	Pacific oyster
**47**	A11330-1 (PFR25D04)	4108	2648	Mahurangi Harbour	NZ	2010	Pacific oyster
**48**	A11331-1 (PFR27A01)	4109	2656	Bay of Islands	NZ	2010	Pacific oyster
**49**	A11332_1 (PFR29A04)	6342	1357	Hauraki Gulf	NZ	2010	Pacific oyster
**50**	A11333_1 (PFR29A05)	6343	378	Hauraki Gulf	NZ	2010	Pacific oyster
**51**	A11335-1 (PFR29A08)	4110	2644	Coromandel	NZ	2010	Pacific oyster
**52**	A11336-1 (PFR29A10)	4111	2653	Kaipara Harbour	NZ	2010	Pacific oyster
**53**	A11338_1 (PFR29B07)	6346	1140	Hauraki Gulf	NZ	2010	Pacific oyster
**54**	A11339_1 (PFR29B08)	6347	1357	Hauraki Gulf	NZ	2010	Pacific oyster
**55**	A11340-1 (PFR29C03)	4112	2649	Hauraki Gulf	NZ	2010	Pacific oyster
**56**	A11341_1 (PFR29C05)	6349	1357	Coromandel	NZ	2010	Pacific oyster
**57**	A11342_1 (PFR29C06)	6350	1357	Mahurangi Harbour	NZ	2010	Pacific oyster
**58**	A11343 (PFR29C09)	4130	2664	Bay of Islands	NZ	2010	Pacific oyster
**59**	A11344_1 (PFR29E07)	6352	2324	Coromandel	NZ	2011	Pacific oyster
**60**	A11345_1 (PFR29F04)	6353	378	Hauraki Gulf	NZ	2011	Pacific oyster
**61**	A11346_1 (PFR29F06)	4082	2636	Mahurangi Harbour	NZ	2011	Pacific oyster
**62**	A11347_1 (PFR29F07)	6355	378	Kaipara Harbour	NZ	2011	Pacific oyster
**63**	A11349-1 (PFR29G10)	4083	2637	Coromandel	NZ	2011	Mussels
**64**	A11350-1 (PFR29H02)	4113	2649	Hauraki Gulf	NZ	2011	Pacific oyster
**65**	A11351-1 (PFR29H05)	4114	2648	Bay of Islands	NZ	2011	Pacific oyster
**66**	A11352_1 (PFR29I01)	6359	378	Hauraki Gulf	NZ	2011	Pacific oyster
**67**	A11353-1 (PFR29I02)	4115	2648	Hauraki Gulf	NZ	2011	Pacific oyster
**68**	A11354_1 (PFR29I09)	6361	378	Kaipara Harbour	NZ	2011	Pacific oyster
**69**	A11355_1 (PFR30A06)	6362	378	Marlborough	NZ	2011	Mussels
**70**	A11356-1 (PFR30B08)	4084	2638	Kaipara Harbour	NZ	2011	Pacific oyster
**71**	A11357_1 (PFR30C07)	6364	1328	Bay of Islands	NZ	2011	Pacific oyster
**72**	A11358_1 (PFR30D03)	6365	1357	Hauraki Gulf	NZ	2011	Pacific oyster

Table S2. Continued.

#	Isolate name	PubMLST accession number	ST	Geographic location	Country	Isolation year	Source
**73**	A11359-1 (PFR30F09)	4085	2631	Hauraki Gulf	NZ	2011	Pacific oyster
**74**	A11360_1 (PFR30G02)	6367	1772	Whangaroa Harbour	NZ	2011	Pacific oyster
**75**	A11361_1 (PFR30G05)	4116	2652	Bay of Islands	NZ	2011	Pacific oyster
**76**	A11362_1 (PFR30G10)	4117	2650	Hauraki Gulf	NZ	2011	Pacific oyster
**77**	A11363_1 (PFR30I02)	4118	2650	Hauraki Gulf	NZ	2011	Pacific oyster
**78**	A11366 (PFR31D06)	4131	2665	Hauraki Gulf	NZ	2011	Pacific oyster
**79**	A11367_1 (PFR31E07)	6372	1357	Whangaroa Harbour	NZ	2011	Pacific oyster
**80**	A11368-1 (PFR32D08)	4086	2639	Whangaroa Harbour	NZ	2011	Pacific oyster
**81**	A11369-1 (PFR32E06)	4087	2631	Bay of Islands	NZ	2011	Pacific oyster
**82**	A11370_1 (PFR34B02)	4119	2648	Coromandel	NZ	2012	Pacific oyster
**83**	A11371_1 (PFR37C05)	6376	1140	Kaipara Harbour	NZ	2012	Pacific oyster
**84**	A11372_1 (PFR37C06)	4120	2650	Kaipara Harbour	NZ	2013	Pacific oyster
**85**	A11373_1 (PFR37D03)	4121	2657	Kaipara Harbour	NZ	2013	Pacific oyster
**86**	A11374_1 (PFR37D08)	4122	2658	NZCC NZRM	NZ	2013	Clinical
**87**	A11375_1 (PFR37E01)	6379	17	NZCC NZRM	NZ	1973	Clinical
**88**	A11376_1 (PFR37E03)	6380	1140	NZCC NZRM	NZ	1975	Clinical
**89**	19ER2032	6232	36		NZ	2019	Clinical
**90**	19ER2175	6233	36		NZ	2019	Clinical
**91**	19ER2200	6234	36		NZ	2019	Clinical
**92**	19ER2252	6381	36		NZ	2019	Clinical
**93**	19ER2253	6235	36		NZ	2019	Clinical
**94**	19ER2263	6236	36		NZ	2019	Clinical
**95**	19ER2264	6237	36		NZ	2019	Clinical
**96**	19ER2305	6238	36		NZ	2019	Clinical
**97**	19ER2454	6240	36		NZ	2019	Clinical
**98**	19ER2456	4073	2630		NZ	2019	Environmental
**99**	19ER2457	6242	1357		NZ	2019	Environmental
**100**	19ER2458	6243	648		NZ	2019	Environmental
**101**	19ER2370	6239	50		Fiji	2019	Clinical
**102**	19ER4464	6244	50		Fiji	2019	Clinical
**103**	20ER0396	6245	50		NZ	2020	Clinical
**104**	20ER0397	6244	50		NZ	2020	Clinical
**105**	20ER0715	6247	50		NZ	2020	Clinical
**106**	20ER0927	6248	50		NZ	2020	Clinical

Table S2. Continued.

#	Isolate name	PubMLST accession number	ST	Geographic location	Country	Isolation year	Source
**107**	20ER0928	6249	50		NZ	2020	Clinical
**108**	20ER0962	6250	50		NZ	2020	Clinical
**109**	20ER0990	6251	50		NZ	2020	Clinical
**110**	20ER1681	6252	50		NZ	2020	Clinical
**111**	20ER1728	6253	50		NZ	2020	Clinical
**112**	20ER1729	6254	50		NZ	2020	Clinical
**113**	20ER1730	6255	50		NZ	2020	Clinical
**114**	20ER1748	6256	50		NZ	2020	Clinical
**115**	20ER1749	6257	50		NZ	2020	Clinical
**116**	20ER1750	6258	50		NZ	2020	Clinical
**117**	20ER1772	6259	50		NZ	2020	Clinical
**118**	20ER1816	6260	50		NZ	2020	Clinical
**119**	20ER1817	6261	50		NZ	2020	Clinical
**120**	20ER1818	6262	50		NZ	2020	Clinical
**121**	20ER1842	6263	50		NZ	2020	Clinical
**122**	20ER1852	6264	50		NZ	2020	Clinical
**123**	20ER1862	6265	50		NZ	2020	Clinical
**124**	20ER2020	6266	50		NZ	2020	Clinical
**125**	20ER2114	6267	50		NZ	2020	Environmental
**126**	20ER2115	6268	50		NZ	2020	Environmental
**127**	20ER2116	6269	50		NZ	2020	Environmental
**128**	20ER2208	6270	50		NZ	2020	Clinical
**129**	20ER3249	6271	50		NZ	2020	Clinical
**130**	21ER0224	6272	50		NZ	2021	Clinical
**131**	21ER0459	6273	50		NZ	2021	Clinical
**132**	21ER0509	6274	50		NZ	2021	Clinical
**133**	21ER0725	6275	50		NZ	2021	Clinical
**134**	21ER0754	6276	199		NZ	2021	Clinical
**135**	21ER0755	6277	199		NZ	2021	Clinical
**136**	21ER0811	6278	50		NZ	2021	Clinical
**137**	21ER0824	6279	50		NZ	2021	Clinical
**138**	21ER0825	6280	50		NZ	2021	Clinical
**139**	21ER0826	6281	50		NZ	2021	Clinical
**140**	21ER0868	6282	50		NZ	2021	Clinical
**141**	21ER0942	6283	50		NZ	2021	Clinical
**142**	21ER0952	6284	50		NZ	2021	Clinical

Table S2. Continued.

#	Isolate name	PubMLST accession number	ST	Geographic location	Country	Isolation year	Source
**143**	21ER0953	6285	50		NZ	2021	Clinical
**144**	21ER0954	6286	50		NZ	2021	Clinical
**145**	21ER0955	6287	50		NZ	2021	Clinical
**146**	21ER1334	6288	50		NZ	2021	Environmental
**147**	21ER1438	6289	50		NZ	2021	Clinical
**148**	21ER1445	6290	50		NZ	2021	Clinical
**149**	21ER1446	6291	50		NZ	2021	Clinical
**150**	21ER1500	6292	36		NZ	2021	Clinical
**151**	21ER3492	6293	199		NZ	2021	Clinical

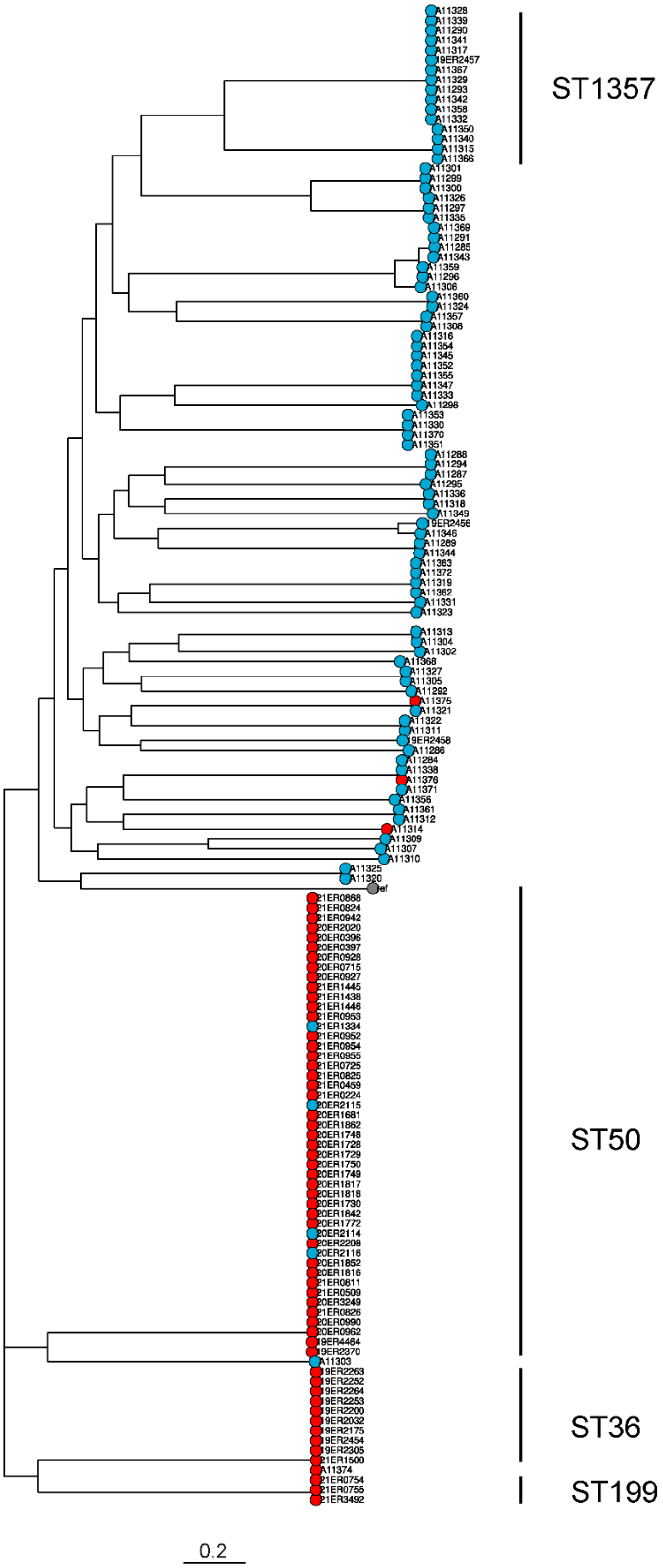

Figure S1. **Phylogeny of clinical and environmental isolates of *V. parahaemolyticus* obtained from New Zealand**. Each isolate was annotated with its sequence type. The population of clinical and environmental isolates is coded with red and blue colors, respectively. Clinical isolates of ST36, ST50 and ST199 and environmental ST1357 were indicated on far right.
